# Importance of Patient Involvement in Creating Content for eHealth Interventions: Qualitative Case Report in Orthopedics

**DOI:** 10.2196/39637

**Published:** 2022-11-03

**Authors:** Thomas Timmers, Walter van der Weegen, Loes Janssen, Jan Kremer, Rudolf Bertijn Kool

**Affiliations:** 1 IQ healthcare Radboud Institute for Health Sciences Radboud University Medical Center Nijmegen Netherlands; 2 Interactive Studios Den Bosch Netherlands; 3 Anna Hospital Geldrop Netherlands; 4 Maxima Medical Center Veldhoven Netherlands

**Keywords:** eHealth, qualitative research, qualitative, focus group, knee, surgery, feedback, user need, patient need, user centered, content codevelopment, patient involvement, co-design, participatory, app design, mobile health, mHealth, health app, orthopedic, mobile phone

## Abstract

**Background:**

In many industries, collaboration with end users is a standard practice when developing or improving a product or service. This process aims for a much better understanding of who the end user is and how the product or service could be of added value to them. Although patient (end user) involvement in the development of eHealth apps is increasing, this involvement has mainly focused on the design, functionalities, usability, and readability of its content thus far. Although this is very important, it does not ensure that the content provided aligns with patients’ priorities.

**Objective:**

In this study, we aimed to explore the added value of patient involvement in developing the content for an eHealth app. By comparing the findings from this study with the existing app, we aimed to identify the additional informational needs of patients. In addition, we aimed to help improve the content of apps that are already available for patients with knee replacements, including the app our group studied in 2019.

**Methods:**

Patients from a large Dutch orthopedic clinic participated in semistructured one-on-one interviews and a focus group session. All the patients had undergone knee replacement surgery in the months before the interviews, had used the app, and were therefore capable of discussing what information they missed or wished for before and after the surgery. The output was inductively organized into larger themes and an overview of suggestions for improvement.

**Results:**

The interviews and focus group session with 11 patients identified 6 major themes and 30 suggestions for improvement, ranging from information for better management of expectations to various practical needs during each stage of the treatment. The outcomes were discussed with the medical staff for learning purposes and properly translated into an improved version of the app’s content.

**Conclusions:**

In this study, patients identified many suggestions for improvement, demonstrating the added value of involving patients when creating the content of eHealth interventions. In addition, our study demonstrates that a relatively small group of patients can contribute to improving an app’s content from the patient’s perspective. Given the growing emphasis on patients’ self-management, it is crucial that the information they receive is not only relevant from a health care provider’s perspective but also aligns with what really matters to patients.

**Trial Registration:**

Netherlands Trial Register NL8295; https://trialsearch.who.int/Trial2.aspx?TrialID=NL8295

## Introduction

### Background

In many industries, collaboration with end users is a standard practice when developing or improving a product or service. This process aims to obtain a better understanding of who the end user is and how the product or service could be of added value to him or her. End-user involvement can also be applied in the development of eHealth apps by inviting patients, in addition to health care providers and software developers, to share their thoughts and ideas. Recent research has demonstrated the importance and effectiveness of user involvement, focusing on the design, functionality, usability, security, and privacy of eHealth apps [1-4]. In addition, users are increasingly involved in assessing the readability of the content that has already been created by health care providers and communication professionals [5,6]. Although having readable content is of great importance, it does not ensure that the content aligns with what really matters to the patients. In other words, a patient might easily understand what they are reading, but it still does not inform them about the topics that are important to them.

**Figure 1:**
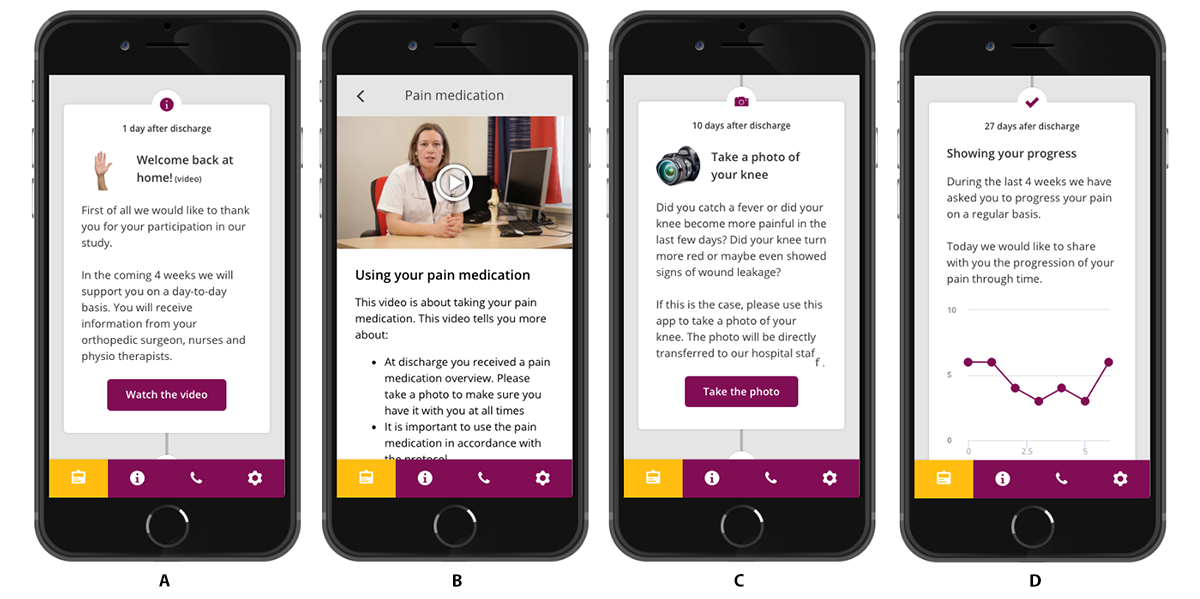
Examples of the interactive app used as an intervention, translated from Dutch (language used in the study) to English. From left to right, (A) the welcoming of patients to the app, (B) video and text information about medication use, (C) an invitation to send a photo of the wound (in case of fever, increased levels of pain, or wound leakage), and (D) a patient-reported pain score–progress tracker.

In 2019, our group studied the effectiveness of an app that provided patients with timely information: small pieces of information that were actively delivered to patients through an app via push notifications when the information was most relevant to patients (Figure 1) [7].

The app we used for the study was developed to support patients undergoing knee replacement surgery, one of the most commonly performed orthopedic procedures worldwide [8-11]. Even though the treatment is highly effective, a 2018 systematic review, including 95,560 patients, indicated that approximately 15% of the patients were unsatisfied [12]. Patients indicate that unfulfilled expectations are the most important reason for this and that improved pre- and postoperative information is needed [13-18]. Previous studies have indicated that the need for improved pre- and postoperative information is not just typical for orthopedic patients [17,19-21]. To develop the content in the 2019 version of the app, we collaborated with orthopedic surgeons, specialized nurses, nurses, and physiotherapists from 5 different Dutch hospitals. In total, the patients received 30 pieces of information and 16 unique videos in a timely manner during the initial months after surgery. Patients were notified of the newly available information through push notifications. Although the results demonstrated significant improvements in outcomes such as pain management, knee function, quality of life, and satisfaction, it is unknown whether the information presented in the app aligns with what really matters to patients, as it was developed solely by health care providers.

To gain an in-depth understanding of additional or different informational needs of patients, firsthand perspectives are required. To obtain these insights, a qualitative research approach is most appropriate, as it allows access to the thoughts and feelings of patients’ understanding of how they have experienced their treatment and how the information that they were offered supported them [22].

### Objectives

In this study, we aimed to explore the added value of patient involvement in developing the content for an eHealth app. By comparing the findings from this study with the existing app, we aimed to identify the additional informational needs of patients. In addition, we aimed to help improve the content of the apps that are already available for patients with knee replacements, including the app our group studied in 2019.

## Methods

### Study Design

This qualitative, mixed methods, prospective cohort study was conducted at the orthopedic department of the St. Anna General Hospital (Geldrop, the Netherlands). The hospital participated in the 2019 study and currently offers this app as the standard of care to patients undergoing knee replacement surgery. As of April 20, 2022, the app was downloaded 2923 times and used more than 120,000 times by patients during various stages of their treatment.

To identify the themes that are significant to most patients, we used a 2-step approach. First, we conducted face-to-face, semistructured interviews with individual patients. After transcribing, coding, and thematizing the results, we invited all the patients to discuss the themes that were identified and the suggestions for improvement that were derived from the interviews during a focus group session. In the final overview of suggestions, we added the element of timing to ensure that the patients receive information when it is most relevant to them. For the duration of the study, the patients were not informed that the members of the research team were involved in the development of the app and its content, as this might have impacted the thoughts and feelings the patients wanted to share, especially with regard to questions about communication from the hospital.

The study was registered at the Netherlands Trial Register (NL8295). Patients were asked to consider study participation by their physiotherapists, specialized nurses, or orthopedic surgeons. Typical sampling was used to ensure that the study population had characteristics similar to those of the general patient population (with respect to age, sex, and marital status). The senior researcher from the hospital (WvdW) contacted the patients who considered participating by phone in the days following surgery. After providing information about the study, a consent form was sent by email. After a few days, the researcher again contacted the patients to answer any remaining questions and to schedule a face-to-face interview with those who were interested in participation. The patients were informed that they would be invited to a focus group session at a later stage of the study. Participants could choose to have their first interview at home or at the hospital. After interviewing a patient at home and a patient at the hospital in February 2020, we were restricted to meetings on the web because of the COVID-19 pandemic. WhatsApp Messenger (Meta) was used to conduct the remaining interviews via video.

We followed the consolidated criteria for reporting qualitative research guidelines to obtain results [23].

### Ethics Approval

Ethics approval was obtained from the Radboud Academic Medical Center Regional Ethics Board (reference 2020.6087) as well as from the St. Anna Local Ethics Board (reference 2020.004).

### Participant Selection

Patients who underwent knee replacement surgery were invited to participate in the study. To limit the risk of recall bias, patients were only eligible for inclusion if they were able to schedule the interview within a maximum of 20 weeks after they had undergone their surgery.

### Interviews

Patient characteristics were collected at the beginning of the interview. Next, an interview topic guide was used to address 3 specific aspects of the perioperative and early postoperative periods (Multimedia Appendix 1). The first specific aspect was general recovery. For this, the topic guide began with open, broad questions about how the patient had experienced recovery so far and which experiences were positive or negative. For each experience recalled, participants were asked to elaborate in as much detail as possible. Specifically, the interviewer asked how the experience was related to what they expected and what the basis was for these expectations. The second aspect was the information they received from their health care providers about their knee replacement recovery and how they aligned with their lived experiences. When a difference or gap was identified between the patients’ expectation and experience, the patients were asked to elaborate on this in as much detail as possible. The third and final aspect of the interview focused on the information and education patients obtained for themselves (eg, consultation with health care providers, brochures, websites or apps, search engines, and social media). When all the 3 aspects were covered, patients were invited to follow-up with information about specific topics or add anything else that they felt was valuable.

A trained qualitative interviewer (TT) conducted the interviews together with a senior orthopedic researcher (WvdW). When the interviewers presumed data saturation and no new insights were gained, 2 additional interviews were conducted to finally evaluate the interviews. All the interviews were audio recorded and professionally transcribed verbatim.

### Focus Groups

After transcribing, coding, and assigning themes to the results, patients were invited to a focus group session on September 24, 2020. The session was held at St. Anna Hospital and lasted from 7:30 PM to 9:15 PM. The session was hosted by TT and WvdW, who were accompanied by an orthopedic physician’s assistant from the hospital (Ellis Bos) to answer the medical questions. A slide presentation served as the topic guide for the session (Multimedia Appendix 2).

To ensure that the session was open to active participation, the hosts began by asking the patients which themes they thought would (or should) be discussed. Next, the research team shared some of the quotes provided during the interviews, allowing participants to define the underlying theme themselves. After participants shared their thoughts and feelings related to the theme, the researchers summarized their findings and provided an overview of the suggestions for improvement that came from the interviews. Patients were invited to provide feedback and add other elements to the overview. Next, a summary was provided, and conclusions were drawn from the results of the interviews and focus group session. Finally, patients were thanked for their participation and received a gift card.

### Data Analysis

The transcription and coding of the interviews commenced after data saturation was reached. All interview transcripts were anonymized and uploaded to Atlas.ti (version 8.4.4; ATLAS.ti GmbH). Two members of the research team (TT and WvdW) independently read the transcripts from the first 3 interviews to identify codes. The researchers then compared their findings and agreed on a coding framework that could be used to code the remaining data. After all the interviews were coded by both researchers, they inductively organized the codes into larger categories using thematic analysis [24]. Finally, the relationships among the themes were identified, and the order in which they would be presented was determined.

## Results

### Study Sample

A total of 11 participants were interviewed between February 2020 and April 2020. The mean age of the patients was 66 (range 57-74) years (Multimedia Appendix 3). In total, 64% (7/11) of women and 36% (4/11) of men were interviewed. The mean duration of the interviews was 13 weeks after surgery (range 10-17 weeks). Before the interview, 18% (2/11) of patients underwent knee replacement surgery of the other knee. An interview was conducted at a patient’s home and another at the hospital, and WhatsApp Messenger was used in 82% (9/11) of cases. The mean duration of the interviews was 40 minutes (range 25-57 minutes).

The following paragraphs provide an overview of the themes that emerged from the interviews. A narrative synthesis and an overview of suggestions for improvement are provided for each theme. In addition, a narrative synthesis is provided of the outcome of the focus group session, as well as the feedback that health care providers provided after the research team had shared their findings with them. Finally, examples of newly implemented information in the app are presented.

### Theme 1: Expectations Versus Reality

A variety of answers were provided when the participants were asked how they experienced their recovery. In most cases, the recovery took longer than expected. Patients often referred to “the first 6 weeks” because they understood that most of the troubles and difficulties with pain, sleeping, and moving around would cease or be minimized by then. These sentiments were evident from the following excerpts from interviews:

My orthopedic surgeon told me that I would be able to get rid of the first crutch after 4 weeks and the second one after 6 weeks. Other people I talked to confirm this. I have to be honest, I guess I also heard the things I wanted to hear.

PT08, female, aged 72 years

I wouldn’t be able to tell you what went well so far. At the moment, my knee hurts more than before the surgery, which was performed 10 weeks ago. I would have thought to have gained quite a lot in the first 6 weeks already.

PT07, female, aged 66 years

It was really 200% better than I expected... I started working behind the bar at the elderly center again about 8 weeks after surgery. I actually wanted to start again after 4 weeks, but my son and my GP wouldn’t allow me. Unfortunately, the whole COVID-19 situation required us to close the bar, but I was up for it.

PT04, female, aged 74 years

Two suggestions for improvements were identified for this theme (Table 1).

**Table 1:** Improvement of information and timing for the theme “Expectations and Recovery.”

Information	Timing
Duration and intensity of the recovery. It takes months, not weeks.	During decision-making for TKR^a^ treatment and as a reminder in the weeks before and the weeks after surgery
Recovery differs greatly from patient to patient. There is no definition or standard for patients to compare themselves with in terms of speed and of duration of recovery.	First week after surgery or repeat 1 or 2 times in the following weeks

^a^TKR: total knee replacement.

### Theme 2: Postoperative Pain

Patients identified postoperative pain as the central theme of early postoperative recovery period. In most patients, the severity and duration of the pain were unexpected. Pain during the night was an unpleasant surprise to many patients, as they expected that pain medication and lying at rest in their bed would provide them with a good night’s sleep. Finally, some patients mentioned being somewhat surprised by the existence and duration of neuropathic pain, ranging from shooting pain throughout the leg to the feeling of a very tight band around the knee as seen in the following quotes:

My orthopedic surgeon had clearly told me that after the surgery it would start to hurt even more and then get better, he was completely right.

PT05, female, aged 61 years

Sufficient attention is paid to the pain in the first few days, but the long-term pain rarely mentioned.

PT08, female, aged 72 years

The first 5 weeks after the operation I slept really bad. It is as if you don’t really feel the pain during the day, but you do at night.

PT11, male, aged 72 years

Six suggestions for improvements were identified for this theme (Table 2).

**Table 2:** Improvement of information and timing for the theme “Postoperative Pain.”

Information	Timing
In the initial weeks or months after surgery, patients will possibly experience more (but different) pain than what they did before the surgery.	After surgery (repeat several times)
Pain during the night is common in patients with knee replacements. This is unexpected, as patients expect the combination of pain medication and lying at rest to be beneficial. Patients might sleep poorly for weeks or even months. This is caused not only by unexpected turning while sleeping but also because of jolting neuropathic pain, the feeling of a tight bandage being wrapped around the knee, or the knee becoming suddenly very warm. Multiple nights of poor sleep can have a serious impact on a patient’s mental and physical well-being, negatively influencing their recovery.	After surgery (repeat several times)
It is advised to use the pain medication as prescribed by the patient’s health care professional. There is a very low risk of addiction to high-dose (rescue) pain medication even when it is only used for a short period.	Initial weeks after surgery
Some patients report side effects from the pain medication. If this is the case, patients should contact the hospital to see if the medication can be changed.	Initial weeks after surgery
For unknown reasons, pain medication is not always effective. Patients should contact the hospital when they feel the medication is not effective, leaving them with unmanageable postoperative pain. The hospital staff might be able to prescribe different, more effective medication.	Initial weeks after surgery
Neuropathy, or nerve pain, is caused by tiny nerves that were damaged or pinched during the surgery. This can lead to a constant or jolting pain around the knee or in the entire leg in the weeks following surgery. Although it might take a long time, in most cases the neuropathy disappears over time.	Initial weeks after surgery; reminder after 1 and 2 months

### Theme 3: Information and Educational Materials

There were various patient needs concerning the type and amount of information they wanted or expected to receive about the treatment. Some patients wanted to know all the details about the surgery and prosthesis, whereas others preferred not to know about the details at all. Patients said that regardless of the type or amount of information they received, it was still very difficult to prepare for something they had never previously undergone. Remarkably, this was also reported by 2 patients who underwent surgery on their other knee <2 years before.

In preparation for their surgery, the patients were advised and supported by the hospital staff to download the hospital’s app for knee replacement surgery. All the patients reported that they had downloaded and used them. Approximately half of the patients enabled the push notification feature from the app and were satisfied push notification feature. The other patients said they were not aware of this feature but still used the app multiple times during their treatment. The videos in the app were often mentioned as a great service, making the information easily available and digestible. Participants felt that because the videos were made by hospital staff, they were a trustworthy source of information. In addition to the app, the patients were offered hospital brochures. Most patients reported that they used the brochures at least once.

Some patients reported that the app should not only focus on complications, pain, and functional outcomes such as bending and stretching of the knee but also practical information, including details about “normal” recovery trajectory as well as the fact that recovery experience differs from patient to patient. The timing of the information in the app did not always align with the patients’ actual recovery time, which unsettled the patients who recovered more slowly.

Patients did not spend much time searching for additional information on the internet. The following excerpts reveal the patient response to educational materials:

I read the app completely, I really liked that. When I received a push notification, I read the information right away.

PT03, female, aged 67 years

You don’t really know what’s normal. You won’t find those feelings anywhere. What am I supposed to feel now? Is this normal? That is of course something very personal, I understand that.

PT09, male, aged 57 years

Four suggestions for improvements were identified for this theme (Table 3).

**Table 3:** Improvement of information and timing for the theme “Informational and Educational Materials.”

Information	Timing
Provide comprehensive, though subdivided information about the anatomy of the knee, origin of complaints, knee replacement components, and the surgery. Ensure patients have a choice for the level of detail, for instance through “read more” links.	At the start of using the app
The app uses push notifications to actively inform patients about newly available information. Advise them to check whether they have enabled this functionality by going to the settings screen of their smartphone or tablet.	At the start of using the app
The recovery trajectory differs from person to person. There is no “normal” recovery or a graph that patients can or have to compare themselves with.	Initial weeks after surgery
The timing of the information in this app might differ from an individual patient’s recovery time; state clearly this is not a cause for concern	Initial weeks after surgery

### Theme 4: Physiotherapy Exercises

The patients unanimously agreed on the importance of physiotherapy sessions after discharge from the hospital. Being with their physiotherapist motivated them to perform the exercises and persuaded them to comfortably bend and stretch their knees just a little more than they would do at home by themselves. In addition, many considered the physiotherapist to be a personal coach. However, some found it confusing that sometimes, even within a single practice, the therapist’s advice on the type of exercises or how to execute them differed from or even contradicted the previous advice. Several patients reported that they performed specific knee strengthening exercises in preparation for their surgery and felt that this benefited them during recovery.

Patients reported various physiotherapy rehabilitation approaches. There were differences not only between group sessions and individual coaching but also in the therapy itself, ranging from “fixing the knee to a bench and applying brute force to it for a short period of time” to sessions where patients would just come in during the day (unscheduled) and spend up to 2 or 3 hours doing semisupervised exercises. When asked about the physiotherapy exercises performed in the hospital directly after surgery, more than one participant mentioned that they did not feel ready for it, stating that it felt a bit hurried and rather strange doing exercises so soon after surgery with a “fresh, 30-cm-long wound” in their knee. Patients voiced their opinion as follows:

I have to say I really need the physiotherapist, because he keeps on saying everything will be fine.

PT07, female, aged 66 years

What is actually rarely mentioned, is the importance of the therapy. The entire course of your rehabilitation depends on how good the therapy and the therapist are.

PT09, male, aged 57 years

What amazes me a bit, and I’m sure they all mean it well, is that one therapist says: “You have to jump to the left” and then the other comes in and says: “What are you doing? You have to jump to the right.”

PT10, male, aged 57 years

Eight suggestions for improvements were identified for this theme (Table 4).

**Table 4:** Improvement of information and timing for the theme “Physiotherapy Exercises.”

Information	Timing
Performing exercises in the 4 to 8 weeks before surgery could strengthen the muscles around the knee and increase the flexibility of the knee.	4 to 8 weeks before surgery
Physiotherapy is offered many ways, ranging from individual to group therapy sessions, and from high-intensity shorter training sessions to longer semisupervised sessions. Regardless of these different forms of physiotherapy, the physiotherapist is seen as someone who motivates and persuades patients to do their exercises (to the full extent). In addition, patients regard their therapist as their personal coach, which is important in difficult times during recovery.	4 to 8 weeks before surgery
It is important for patients to find a physiotherapy practice that matches their personal preferences. Visiting clinics before the surgery could help in finding the right practice.	4 to 8 weeks before surgery
Before surgery, patients can start practicing walking, getting in and out of a chair, and climbing stairs with crutches. This will make the patient more comfortable using crutches when they return home from the hospital.	2 to 4 weeks before surgery
Patients should be aware of the fact that almost directly after the surgery, they will start to perform physiotherapy exercises with one of the hospital’s physiotherapists. This might feel as something impossible to do right after surgery, but many patients are not aware that rapid mobilization of the knee has a positive impact on the progress of the recovery.	1 week before surgery
Patients should be aware of the importance of moving around and performing exercises. Even though it might sound or feel strange after undergoing major surgery, mobilization of the knee is of vital importance for a successful recovery. It is recommended to share the rationale behind early mobilization with patients after surgery and before performing the exercises.	Initial weeks after surgery
Patients should contact their physiotherapist when the instructions they receive are unclear or seem contradictory.	Initial weeks after surgery
There are major differences in recovery among patients. For example, the distance people can walk or cycle without too much pain, stiffness, or swelling of the knee varies widely. Patients should bear in mind that each recovery trajectory is very unique and that patients should not always compare their experience with others’ or even with their own previous joint replacement surgery experience if they had one.	Initial weeks after surgery
Severe postoperative stiffness of the knee is, to a certain extent, something that can be treated through physiotherapy exercises. Only in rare cases patients are admitted to the hospital again, where the orthopedic surgeon manipulates the knee under local anesthesia to improve the knee’s range of motion. This procedure is effective but adds weeks to months to the recovery trajectory.	4 to 8 weeks after surgery

### Theme 5: Activities of Daily Living

Major differences in activities of daily living were noted among the patients. A patient stated that he was disappointed that 6 weeks after surgery, he was able to walk for only 200 m. Another patient reported being able to walk for approximately an hour, 6 weeks after surgery but was still rather disappointed because she expected to be able to do more. In addition, most patients mentioned having trouble cycling and walking. In many cases, this led to increased knee stiffness and swelling as did walking stairs, and one patient described it as, “dragging yourself up the stairs and jumping down the stairs, just to prevent the knee from bending.” Taking a shower was another example in which clinical guidelines and patient experiences contradicted. From a clinical perspective, it was safe to take a shower, but from a practical perspective, it took patients about an hour and a half to do so, exhausting them. Three additional reported issues were sitting on the toilet (and getting up), difficulty walking with crutches, and the use of a walker or rollator to support in-house activities such as getting a cup of coffee and carrying small items through the house. A patient wore an Apple Watch (Apple) for fall detection because she lived alone. This technology allowed her neighbors to be notified if she had fallen. The following quotes highlight the reported issues:

Yes, I can cycle. Distances of just 20 or 30 km because my physical condition has deteriorated a bit.

PT04, female, aged 73 years

When I get on the exercise bike in the morning, I do a few cycles forwards and backwards. Then, I can cycle for 10 to 12 mins. The second time it takes a little longer to prepare, and in the evening, my knee is really stiff and thick.

PT06, female, aged 72 years

Nine suggestions for improvements were identified for this theme (Table 5).

**Table 5:** Improvement of information and timing for the theme “Activities of Daily Living.”

Information	Timing
By using a toilet seat riser, patients will not have to bend their knees to the extent they would with a standard, much lower, toilet seat. This improves patient comfort in the postoperative phase.	2 to 4 weeks before surgery
Even though using crutches in the initials weeks after surgery enable mobility, carrying things such as coffee or lunch from one place to another with crutches is almost impossible. Using a walker or rollator may help patients manage their own health.	2 to 4 weeks before surgery
Instant discoloring of the knee or entire leg may appear in the initial weeks after surgery. This is something that can happen overnight, ranging from yellow green to dark blue and purple. This is something patients should not worry about, and it often disappears within several weeks.	Initial weeks after surgery
For some patients, it might be more comfortable to use a patch or band-aid on the knee to prevent clothing from continuously rubbing over the wound on the knee.	Initial weeks after surgery
Wearing soft clothing, such as sweat pants or training pants, protects the knee and the wound from uncomfortable pressure and rubbing.	Initial weeks after surgery
The knee might stiffen after surgery, which can last for weeks or even months. The knee can be stiff when getting out of bed in the morning or become stiff (again) during the day after activity. Specific exercises or training on a home trainer for 10 to 15 minutes helps in the prevention of stiffness.	Initial weeks after surgery
Swelling of the knee is reported by many patients, and it can last for weeks or even months. Swelling of the knee often occurs during or after activities such as walking or cycling. When the knee is swollen, patients are advised to elevate the leg (place pillow under the calf, not the knee itself) and apply ice packs on the knee.	Initial weeks after surgery
Taking a shower is often allowed from the moment patients get home. However, patients should be told explicitly that oftentimes, going up and down the stairs, undressing and dressing, and taking a shower are not very easy after knee replacement surgery. It will take a very long time and can be exhausting. It is advised to place a high stool in the shower to rest upon. Ensure the stool is stable and cannot slip or slide away.	Initial weeks after surgery
Patients indicate that in many cases, ointment with vitamin E helps to alleviate knee pain or itchiness.	From 2 weeks after surgery onwards

### Theme 6: Hospitalization and Aftercare

All the patients reported that they felt very welcome at the hospital and that they were supported from arrival onwards by very friendly and helpful hospital staff, who had time to talk to them and answer their questions. For some patients, the discharge process felt a bit hurried, such as finishing a checklist, but no one felt as if they left the hospital without having enough information to take care of themselves. The fact that most patients left the hospital after only 24 hours still amazed some of them, mainly because they had undergone a major surgery. Other patients were more than happy to go home and were confident that being at home would be beneficial for their recovery.

Approximately 1 week after the surgery, all the patients received a phone call from one of the specialized orthopedic nurses, regardless of whether or not they had contacted the hospital themselves. Although clearly stated in the information patients received from the hospital, the phone call came as a pleasant surprise to patients, as it allowed them to ask questions or just briefly share their experiences. Patients who initiated phone calls or extra hospital visits were also very positive about the experience. This was not always the case with the formal 8-week follow-up consultation, which was reported by some patients to be rather clinical as it focused on the x-ray results. The consultation focused on discussing the x-ray results, that pain is normal, and that performing exercises benefits the recovery. The patients reported that they expected to have more meaningful and practical information about their recovery during this consultation as follows:

I thought it was great from the moment of admission onwards. In the operating room and the recovery room too, that all went perfectly.

PT02, male, aged 62 years

After about a week, I also got a call (from one of the nurses). I could talk a little about medication. The person you are talking to is really someone who understands what you are going through.

PT03, female, aged 67 years

Two suggestions for improvements were identified for this theme (Table 6).

**Table 6:** Improvement of information and timing for the theme “Hospitalization and Aftercare.”

Information	Timing
When patients are informed about receiving a phone call from a health care provider, they are advised to write down their questions in advance.	Initial days after surgery (depending on when the call is)
Patients might be informed that the follow-up consultation with their orthopedic surgeon is focused on the alignment of the knee prosthesis, the pain, and importance of exercises in the coming months. Patients might be advised to write down their questions in advance.	Approximately a week before the follow-up consult

### Results From the Focus Group Session

All 11 patients were invited to participate in the focus group session; 7 (64%) of them accepted the invitation. Owing to mandatory self-quarantine and COVID-19–related symptoms, 29% (2/7) of patients had to cancel their participation, and 71% (5/7) of participants were finally included. The focus group session led to new or additional participant insights in some cases; however, in most cases, the findings of the research team were confirmed. One of the main discussion items was why, from a medical perspective, there were differences among patients regarding pain management, duration of recovery, or recommendations for returning to sports, hobbies, or work. Ellis Bos and WvdW responded that both from a clinical and scientific perspective, these differences had been reported before and that it was still unclear why they occurred. The differences in recovery reported by the 2 patients who underwent total knee replacement in both knees were exemplary for this phenomenon.

At the end of the focus group session, the research team provided an overview of the suggestions for each theme to improve the current educational materials for patients undergoing knee replacement surgery. Feedback on these suggestions and additional ideas were discussed, leading to a final overview of the improvements.

### Health Care Providers’ Response to the Feedback From Patients

Findings from the interviews and focus group session were shared with the health care providers at the orthopedic department. Their first response was a mixture of being glad that the study was performed because it provided new insights into what matters to patients and being surprised that so much of the information that they normally shared with patients did not resonate. This ranged from complex topics such as the duration of the recovery phase and pain management in the early postoperative phase to practical measures such as using toilet seat raiser or knowing how to act when the knee starts to swell. The difference between clinically oriented information and practical patient perspectives was best described by the example of “taking a shower.” which is okay from a clinical perspective but considered a difficult endeavor from a patient perspective. The same goes for climbing up and down the stairs and getting dressed. Finally, the team was positive about the fact that all the patients had arranged their physiotherapy sessions but at the same time, was surprised to hear about the many different types of therapies patients described and the differences in approach, even within the same practice.

### Examples of the Newly Implemented Information in the App

**Figure 2:**
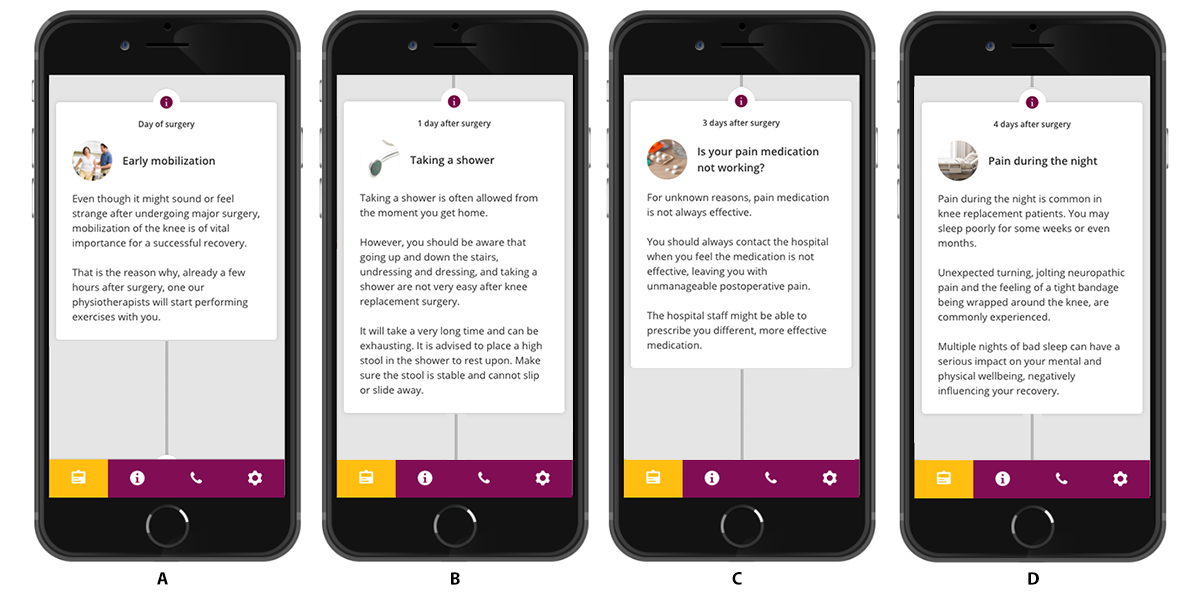
Examples of the information that has been added to the app (translated from Dutch to English). From left to right, (A) early mobilization after surgery, (B) taking a shower, (C) ineffective pain medication, and (D) pain at night.

Some of the suggestions from the interviews and focus group session have already been included in the app that is currently in use at the hospital (Figure 2).

## Discussion

### Principal Findings

The results of our study demonstrate the added value of patient involvement in developing patient-centered content for an eHealth app. From the interviews and focus group session, we learned that health care providers tended to focus on the clinical aspects of recovery such as pain, range of motion, complications, and wound care. However, patients are more interested in practical matters such as the (unexpected) intensity and duration of pain, taking a shower, dealing with swelling, going for a walk, riding a bike, or driving a car. In addition, they want to know “what is normal” in the abnormal situation in which they undergo surgery. Compared with the health care provider–developed content of the existing app for knee replacement patients, more than 30 suggestions for improvement came from patients.

Previous studies, both in orthopedics and other medical fields, have identified patients’ need to improve education and information delivery, to set realistic expectations about recovery period, to improve satisfaction after the treatment, and to increase self-management of pain [3,13-15,17,19,25-28]. However, most studies have not provided practical information on how to achieve and implement these improvements. We aimed to identify these gaps in information for patients, presented them to health care providers, jointly discussed them during a focus group session, and processed the outcomes into a ready-to-use overview and an update of the existing app.

### Limitations

A limitation of our study is the relatively small number of patients who were interviewed. However, even this small group of patients provided valuable insights to improve the existing health care provider–developed content. Moreover, both interviewers agreed that data saturation had already been reached after interviewing 9 patients after which they decided to interview 2 more patients to confirm this. In addition, the inclusion of patients from a single hospital might limit the generalizability of our results. Given that we found more than 30 suggestions for improvement while the participating hospital already offers patients an app, a brochure, a website, and a complimentary postoperative phone call, we are confident that patient involvement in content creation can be of added value for other hospitals as well.

### Clinical Implications and Future Research

Our study demonstrates that patients’ practical informational needs can differ substantially from the clinically oriented information that health care providers want to offer them. The significant question is not whether health care providers offer this information, patients are overwhelmed with information overload, or the timing or format of content delivery was incorrect, but rather what if patients overlooked the information and were therefore unable to use it to set realistic expectations or manage their own health.

When patients and providers collaborate on the development of content, they can learn from each other’s perspectives and create a blend of clinically and practically relevant information. In this process, we suggest inviting patients who having undergone the procedure, provide valuable opinion. Inviting patients from, for instance, a general hospital panel or patient advocate group will ensure the readability of the content and usability of the app, but they cannot determine whether the information itself fits the treatment-specific needs.

Finally, from an implication and implementation perspective, we suggest inviting the (software) developer or developers to participate in the interviews and focus group sessions. The firsthand patient perspectives will teach and inspire them to consider input from end users even more when building new content and software. For future research, we suggest focusing on the effects of patient-centered approach on patient-reported outcomes, patient expectations, contact between the patient and the hospital, and the level of satisfaction with the procedure and recovery.

### Conclusions

This study demonstrates the added value of involving patients when creating the content of eHealth interventions. In total, more than 30 suggestions for improvement were identified, ranging from information to better manage expectations to different kinds of practical needs during various stages of the treatment. In addition, our study demonstrated that a relatively small group of patients can contribute to the improvement of an app’s content. Given the growing emphasis on patients’ self-management, it is crucial that the information they receive is not only relevant from a health care provider’s perspective but also aligns with patient’s priorities.

## Appendix

Multimedia Appendix 1 Semistructured topic guide used for the interviews.

Multimedia Appendix 2 PowerPoint presentation for the focus group.

Multimedia Appendix 3 Participant characteristics.

## Data Availability

Extracts from the data sets used and analyzed during this study are available from the corresponding author upon reasonable request. For confidentiality reasons, the full transcripts cannot be provided.
