# Supplementary material for: Importance of Patient Involvement in Creating Content for eHealth Interventions: Qualitative Case Report in Orthopedics
Source: JMIR Form Res. 2022 Nov 3;6(11):e39637. doi: 10.2196/39637 (PMC9672996; doi:10.2196/39637)
Supplement: Multimedia Appendix 1 [file formative_v6i11e39637_app1.pdf]

Table S1. Semi Structured Interview Topic Guide

Question	Answer
What is your name	
What is your age	
When was your TRK	
What is your home situation	[ ] Living alone<br>[ ] Living together
**Work**	
Did you have a job before the surgery	[ ] No<br>[ ] Yes________________
Have you already (partly) started working again?	[ ] No<br>[ ] Yes
Did you expect this in terms of duration or loading of the knee?	[ ] No<br>[ ] Yes
**Care**	
Were you an informal care giver begore the surgery?	[ ] No<br>[ ] Yes
Have you (party) resumed these activities already?	[ ] No<br>[ ] Yes
Did you expect this in terms of duration or loading of the knee?	[ ] No<br>[ ] Yes
**Hobbies & Sport**	
Did you practice sports of hobbies before the surgery?	[ ] No<br>[ ] Yes
Have you (party) resumed these activities already?	[ ] No<br>[ ] Yes
Did you expect this in terms of duration or loading of the knee?	[ ] No<br>[ ] Yes,
**Recovery**	
How satisfied are you with the recovery so far?	[
What went better than expected?	

What was disappointing so far? E.g. Pain, exercises, wound care, complications, medication usage, lowering medication dose, self care, sleeping, ADL, groceries, showering, walking, cycling, bending the knee, etc.	
Did you, in hindsight, have a realistic view on the recovery trajectory?	
**Information**	
In what way could the hospital have supported you better in terms of information provisioning? (Link these to the topics that were disappointing so far)	
Why does this information matter to you	
At what point in the knee journey would you like to have known about this?	
What sources of information did you use?	
**App**	
Are you familiar with the hospital's app?	
How could they improve the app>	
**End**	
