# Supplementary material for: Importance of Patient Involvement in Creating Content for eHealth Interventions: Qualitative Case Report in Orthopedics
Source: JMIR Form Res. 2022 Nov 3;6(11):e39637. doi: 10.2196/39637 (PMC9672996; doi:10.2196/39637)
Supplement: Multimedia Appendix 2 [file formative_v6i11e39637_app2.pdf]

St. Anna Ziekenhuis

Focus Group Session
Interview Study TKR recovery

24 september 2020

St. Anna Ziekenhuis

Agenda

1. Introduction
2. Backgrounds
3. Aim for this session
4. Results
5. Suggestions for improvement
6. Discussion
7. Conclusion
8. End

St. Anna Ziekenhuis

1. Introduction

- Thomas, Walter, Ellis
- Please introduce yourselves

St. Anna Ziekenhuis

2. Backgrounds

- TKR is major surgery
- Importance of education to manage expectations, to prepare for surgery, to manage yourself after surgery, etc.
- Previously developed app: content developed with healthcare providers

St. Anna Ziekenhuis

2. Backgrounds

- TKR is major surgery
- Importance of education to manage expectations, to prepare for surgery, to manage yourself after surgery, etc.
- Previously developed app: content developed with healthcare providers

- **Where are you? The patients…**
- **Is the information you get enough? Does it cover the themes that matter to you?**

St. Anna Ziekenhuis

2. Backgrounds

- We performed 11 interviews
- Over 9 hours of audio materials
- Transcribed and coded

St. Anna Ziekenhuis

3. Aim

- Do you recognise our summary and findings?
- Did we identify the right themes?
- Did we translate your suggestion into usefull information?

St. Anna Ziekenhuis

3. Aim

- The answer is up to you
- Your opinion is what matters most
- There are no wrong answers
- This is the moment to speak up
- After tonight we plan on implementing the changes in the app

St. Anna Ziekenhuis

4. Results

- Theme by Theme
- Quotes to illustrate the theme
- Summary of findings

St. Anna Ziekenhuis

4. Results

- **Question: which themes do you think we derived from the interviews**

St. Anna Ziekenhuis

"It was really 200% better than I expected… I started working behind the bar at the elderly center again about 8 weeks after surgery.
I actually wanted to start again after 4 weeks, but my son and my GP wouldn't allow me"

"I wouldn't be able to tell you what went well so far. At the moment, my knee hurts more than before the surgery, while it was performed 10 weeks ago. I would have thought to have gained quite a lot in the first 6 weeks already".

St. Anna Ziekenhuis

Guess which theme the quotes belong to…

St. Anna Ziekenhuis

Expectations versus reality

- Major differences in the duration and progression of the recovery

- Only 2 patients are positive, overall the recovery took longer than expected
- Most of you mentioned “the 6 weeks after surgery”, expecting things to improve from that moment onwards

St. Anna Ziekenhuis

"My orthopedic surgeon had clearly told me that after the surgery it would start to hurt even more and then get better, he was completely right"

"After the operation, I was almost unable to sleep. I was used to sleep very well, but now I lay awake all night with a feeling of restlessness in my legs. After a week of bad sleep, you are really exhausted".

St. Anna Ziekenhuis

Guess which theme the quotes belong to…

St. Anna Ziekenhuis

Postoperative Pain

- Pain is a central theme during the recovery
- For most of you, the pain was more intense than expected

- Pain medication did not always work, and some suffered from side-effects

- Pain during the night was highly unexpected
- Numb feeling of the knee or shooting pain thoughout the night

St. Anna Ziekenhuis

"I read the app completely, I really liked that. When I received a push notification, I read the information right away"

"You don't really know what's normal. You won't find those feelings anywhere. What am I supposed to feel now? Is this normal? That is of course something very personal, I understand that"

St. Anna Ziekenhuis

Guess which theme the quotes belong to…

St. Anna Ziekenhuis

Informational and educational materials

- Differences in the level of detail you prefer

- Everyone downloaded the app. Not everyone activated the notifcations.
- Videos were much appreciated
- The app could contain more practical information

St. Anna Ziekenhuis

“What actually remains underexposed is the importance of the therapy. The entire course of your rehabilitation depends on how good the therapy and the therapist are.”

“What amazes me a bit, and I’m sure they all mean it well, is that one therapist says: “You have to jump to the left” and then the other comes in and says: “What are you doing? You have to jump to the right.”

St. Anna Ziekenhuis

Guess which theme the quotes belong to…

St. Anna Ziekenhuis

Physiotherapy exercises

- Everyone mentioned the importance of going to the physio therapist.
- Most of you regard their therapist as a personal coach
- Sometimes physio therapists advices contradict, which is very confusing

- Some of you feel it is rather strange to perform the first exercises directly after surgery

St. Anna Ziekenhuis

“Yes, I can cycle. Distances of just 20 or 30 kilometers because my physical condition has deteriorated a bit.”

“When I get on the exercise bike in the morning, I do a few cycles forwards and backwards. Then, I can cycle for 10 to 12 minutes. The second time it takes a little longer to prepare, and in the evening, my knee is really stiff and thick.”

St. Anna Ziekenhuis

Guess which theme the quotes belong to…

St. Anna Ziekenhuis

Activities of Daily Living

- Major difference after 8 tot 10 weeks: being able to walk for 200 meters or cycly for 20 kilometers
- Cycling is more difficult than expected, often leading to stiffness and swelling of the knee
- Climbing the stairs, taking a shower are regarded as painful

St. Anna Ziekenhuis

“‘ I thought it was great from the moment of admission onwards. In the operating room and the recovery room too, that all went perfectly.”

“After about a week, I also got a call (from one of the nurses). I could talk a little about medication. The person you are talking to is really someone who understands what you are going through.”

St. Anna Ziekenhuis

Guess which theme the quotes belong to…

St. Anna Ziekenhuis

Hospitalization & Aftercare

- Everyone appreciated their stay in the hospital
- Discharge process felt a bit hurried to some of you
- Some thought 1 night in the hospital was to short, other couldn’t wait to get home

St. Anna Ziekenhuis

5. Suggestions for improvement

- Let's look at the hand-outs (these are the overviews we will present in the article, and we will use to optimize the content in the app)

St. Anna Ziekenhuis

6. Discussion

- What do you think of the themes we identified and the suggestions for improvement

St. Anna Ziekenhuis

7. Conclusion

- Do you think that implementing the results from the interviews and this session will lead to an improved version of the app?

St. Anna Ziekenhuis

8. End

- Thank you so much for your personal stories and the contribution to our project
