# Supplementary material for: Importance of Patient Involvement in Creating Content for eHealth Interventions: Qualitative Case Report in Orthopedics
Source: JMIR Form Res. 2022 Nov 3;6(11):e39637. doi: 10.2196/39637 (PMC9672996; doi:10.2196/39637)
Supplement: Multimedia Appendix 3 [file formative_v6i11e39637_app3.pdf]

Table S2. Participant characteristics

**ID**	Sex	Age	Setting	Interview duration (minutes)	Weeks after surgery	Living alone	(Unpaid) work	Sports or hobby	(Informal) care giver
PT01	F	67	WA	47	12	Y	N	Y	N
PT02	M	62	WA	43	10	N	N	N	Y
PT03	F	67	WA	38	9	N	Y	Y	N
PT04	F	73	WA	25	17	N	Y	Y	Y
PT05	F	61	WA	28	16	Y	N	N	N
PT06	F	72	WA	42	13	N	N	N	N
PT07	F	66	WA	30	11	N	Y	Y	Y
PT08	F	72	WA	45	17	N	Y	Y	Y
PT09	M	57	Home	57	15	Y	Y	Y	N
PT10	M	57	Hospital	48	11	N	Y	Y	N
PT11	M	74	WA	41	12	N	N	Y	N
